# Three Novel Entomopathogenic Fungi From China and Thailand

**DOI:** 10.3389/fmicb.2020.608991

**Published:** 2021-01-08

**Authors:** De-Ping Wei, Dhanushka N. Wanasinghe, Jian-Chu Xu, Chaiwat To-anun, Peter E. Mortimer, Kevin D. Hyde, Abdallah M. Elgorban, Sumedha Madawala, Nakarin Suwannarach, Samantha C. Karunarathna, Saowaluck Tibpromma, Saisamorn Lumyong

**Affiliations:** ^1^CAS Key Laboratory for Plant Diversity and Biogeography of East Asia, Kunming Institute of Botany, Chinese Academy of Sciences, Kunming, China; ^2^Department of Entomology and Plant Pathology, Faculty of Agriculture, Chiang Mai University, Chiang Mai, Thailand; ^3^Center of Excellence in Fungal Research, Mae Fah Luang University, Chiang Rai, Thailand; ^4^World Agroforestry Centre, East and Central Asia, Kunming, China; ^5^Centre for Mountain Futures, Kunming Institute of Botany, Kunming, China; ^6^Yunnan Key Laboratory for Fungal Diversity and Green Development, Kunming Institute of Botany, Chinese Academy of Sciences, Kunming, China; ^7^Mushroom Research Foundation, Chiang Mai, Thailand; ^8^School of Science, Mae Fah Luang University, Chiang Rai, Thailand; ^9^Innovative Institute of Plant Health, Zhongkai University of Agriculture and Engineering, Guangzhou, China; ^10^Department of Botany and Microbiology, College of Sciences, King Saud University, Riyadh, Saudi Arabia; ^11^Department of Botany, Faculty of Science, University of Peradeniya, Peradeniya, Sri Lanka; ^12^Department of Biology, Faculty of Science, Chiang Mai University, Chiang Mai, Thailand; ^13^Research Center of Microbial Diversity and Sustainable Utilization, Faculty of Science, Chiang Mai University, Chiang Mai, Thailand; ^14^Academy of Science, The Royal Society of Thailand, Bangkok, Thailand

**Keywords:** Insect fungi, Ophiocordycipitaceae, *Paraisaria alba*, *Paraisaria arcta*, *Paraisaria rosea*, taxonomy, Yunnan Province

## Abstract

Entomopathogenic fungi are ubiquitous in tropical rainforests and feature a high level of diversity. This group of fungi not only has important ecological value but also medicinal value. Nevertheless, they are often ignored, and many unknown species have yet to be discovered and described. The present study aims to contribute to the taxonomical and phylogenetic understanding of the genus *Paraisaria* by describing three new species collected from Guizhou and Yunnan Provinces in China and Krabi Province in Thailand. The three novel species named *Paraisaria alba, P. arcta*, and *P. rosea* share similar morphologies as those in the genus *Paraisaria*, containing solitary, simple, fleshy stroma, completely immersed perithecia and cylindrical asci with thickened caps and filiform ascospores that often disarticulate at maturity. Phylogenetic analyses of combined LSU, SSU, TEF1-α, RPB1, RPB2, and ITS sequence data confirm their placement in the genus *Paraisaria.* In this study, the three entomopathogenic taxa are comprehensively described with color photographs and phylogenetic analyses. A synopsis table and a key to all treated species of *Paraisaria* are also included.

## Introduction

Entomopathogenic fungi are a group of unicellular or multicellular, heterotrophic, eukaryotic microorganisms that can enter into a parasitic relationship with parasitized insects, killing or otherwise disabling their hosts (Samson et al., 1988). They reproduce via sexual or asexual spores, or both (Mora et al., 2017). It is of global importance to survey and describe insect pathogens (Hyde et al., 2019). Entomopathogenic fungi can act as natural enemies of agricultural pests and play an important role in maintaining ecological balance (Fernández-Grandon et al., 2020; Sobczak et al., 2020). For example, fungal pathogens such as, *Coelomomyces, Culicinomyces*, and *Lagenidium* have the capacity to kill larva and adult mosquitoes, reducing their host population (Scholte et al., 2004). Some entomopathogenic fungi, e.g., *Beauveria bassiana, Beauveria brongniartii, Metarhizium anisopliae*, and *Verticillium lecanii*, have been developed as biocontrol agents usable against agricultural pests like aphids, locusts, grasshoppers and cockchafer in Africa and Europe (Roberts and Hajek, 1992; Shah and Pell, 2003). *Beauveria bassiana* and *B. brongniartii* were found to be especially safe bioinsecticides (Zimmermann, 2007). Additionally, some insect pathogens with pharmacological activities are frequently studied, such as *Cordyceps militaris* extract, which exhibits antitumor properties (Li et al., 2020). *Cordyceps* spp. have been utilized as therapeutic agents for metabolic-related disorders (Cao et al., 2020). *Cordyceps cicadae* has renoprotective effects on hypertensive renal injuries (Huang et al., 2020). Entomopathogenic fungi have important biotechnological applications (Hyde et al., 2019) and *Paraisaria* is no exception. Several studies have explored the importance of *Paraisaria* species, such as their antioxidative activity (Ma et al., 2012), nucleoside components (Suo et al., 2013), intracellular polysaccharide composition (Wang et al., 2019) and AGS gastric cancer cells anti-proliferation effects (Ye et al., 2015). Additionally, *P. heteropoda* reportedly produces anti-bacterial and anti-fungal compounds (Krasnoff et al., 2005). Experiments into optimal cultural conditions and nutritional sources were conducted by Sung et al. (2011). Applications of other species in this genus have been poorly studied.

Entomopathogenic fungi are phylogenetically diverse and taxonomically distributed in Ascomycota, Basidiomycota, Chytridiomycota, Entomophthoromycota, Microsporidia, Oomycota and Zygomycota (Vega et al., 2012; Araújo and Hughes, 2016; Mora et al., 2017). Different groups of entomopathogens usually develop respectively unique strategy to colonize their hosts (Mora et al., 2017). It is worth to mention that entomopathogenic taxa in Entomophthorales (Entomophthoromycota) enter into biotrophic relationships with their insect hosts, while those in Hypocreales (Ascomycota) can be hemibiotrophic at earlier stages and transform into saprophytism (Shah and Pell, 2003). The diversity, taxonomy and phylogeny of entomopathogenic fungi have been extensively studied recently (Aung et al., 2008; Mora et al., 2017; Hyde et al., 2018). Most insect pathogens are known from three families: Clavicipitaceae, Cordycipitaceae, and Ophiocordycipitaceae. They are found in the Hypocreales, Hypocreomycetidae, Sordariomycetes, Ascomycota (Sung et al., 2007a; Maharachchikumbura et al., 2016; Wijayawardene et al., 2018). The generic composition of Ophiocordycipitaceae underwent several changes over time (Sung et al., 2007a; Quandt et al., 2014; Maharachchikumbura et al., 2016; Shrestha et al., 2017; Wijayawardene et al., 2018), and currently ten genera are accepted (Hyde et al., 2020). New combinations of these genera were proposed for *Polycephalomyces* by Kepler et al. (2013), *Tolypocladium* by Quandt et al. (2014), *Perennicordyceps* by Matočec et al. (2014) and *Drechmeria*, *Harposporium*, *Ophiocordyceps*, and *Purpureocillium* by Spatafora et al. (2015). The genus *Paraisaria* was recently recovered in Ophiocordycipitaceae (Mongkolsamrit et al., 2019).

The genus *Paraisaria* was established by Samson and Brady (1983), with *P. dubia* as the type species, whose sexual morph was known as *Ophiocordyceps gracilis* (syn. *Cordyceps gracilis*). The sexual morph of this genus is characterized by solitary stromata with a stipe terminating in a globose or ellipsoid fertile head, completely immersed, ostiolate, gregarious perithecia, cylindrical asci and hyaline, filiform, multi-septate ascospores, which break into aseptate fragments when mature. Its asexual morphs are characterized by verticillate branched conidiophores, phialidic, flask-shaped, usually sympodially proliferating conidiogenous cells, which terminate in 1–4 necks, and aseptate, hyaline, smooth-walled conidia, which usually aggregate in slimy heads (Samson and Brady, 1983). Li et al. (2004) synonymized *Isaria gracilioides* under *P. gracilioides* and linked its sexual morph to *Ophiocordyceps gracilioides*. Evans et al. (2010) found the asexual morph of *P. myrmicarum* from a red ant host (*Myrmica rubra*) in a natural environment in the United Kingdom. Quandt et al. (2014) have dropped the genus *Paraisaria* and used its sexual genus *Ophiocordyceps* according to the ‘one fungus one name’ principle. Mongkolsamrit et al. (2019) resurrected *Paraisaria* on the basis of three new species, e.g., *P. orthopterorum*, *P. phuwiangensis*, and *P. yodhathaii* as well as eight new combinations, e.g. *P. amazonica* (Sanjuan et al., 2015), *P. blattarioides* (Sanjuan et al., 2015), *P. coenomyiae* (Ban et al., 2015), *P. gracilioides* (Kobayasi, 1941; Pérez-Villamares et al., 2017), *P. gracilis* (Samson and Brady, 1983; Pérez-Villamares et al., 2017), *P. heteropoda* (Sung et al., 2011; Mongkolsamrit et al., 2019), *P. paramyrmicarum* (= *P. myrmicarum*) (Evans et al., 2010) and *P. tettigonia* (Wen et al., 2016). So far, together with the three new species in this study, 14 species are accepted in *Paraisaria*.

This study is part of a larger survey of fungi in the Greater Mekong Subregion where we came across numerous new taxa (Hyde et al., 2018). In this study, three specimens of entomopathogenic fungi were collected from disturbed forests in China and Thailand, and the typical macro- and micro- morphological characteristics indicate that they are of the *Paraisaria* species. The multigene phylogenetic analysis of LSU, SSU, TEF1-α, RPB1, RPB2, and ITS confirmed their placement within *Paraisaria* as three distinct new species.

## Materials and Methods

### Sample Collection, Isolation, and Morphological Studies

In this study, a total of four fungal specimens were collected. One specimen (HKAS 102484) was collected from Krabi Province in Thailand on an adult cricket. Two specimens (HKAS 102553 and HKAS 102552) on dead larvae of *Lepidoptera* sp. were collected from Guizhou Province of China. One specimen (HKAS 102546) was collected from Yunnan Province in China on *Coleoptera* sp. larva. Among them, the hosts of specimens HKAS 102484, HKAS 102553 and HKAS 102552 were found completely immersed into soil with the stroma protruding from the ground in a forest. Specimen HKAS 102546 was found in a similar condition, but differed in that it was found under a karst stone formation. Macro-morphological characteristics of fresh collections were recorded with a camera (iPhone XS Max) in the field and then the specimens were transported to the laboratory in plastic boxes for subsequent studies. The culture of the specimen HKAS 102546 was created by transferring a small mass of mycelium inside the body of the host into potato dextrose agar (PDA, 1% w/v peptone) using a burned needle and incubated at room temperature (25°C). The pure culture was stored in twice-sterilized water, a 15% glycerinum solution and PDA medium, and deposited in the KUMCC culture collection of the Kunming Institute of Botany (KIB), Chinese Academy of Sciences (CAS). The fruiting bodies were dried with allochroic silica gel and deposited in KUN herbarium of KIB. Facesoffungi numbers were registered as outlined in Jayasiri et al. (2015).

The fresh fruiting bodies were examined and hand-sectioned under an Optec SZ660 stereo dissecting microscope. The key fungal structures viz. ascomata, perithecia, peridium, asci and ascospores were mounted in sterilized water or cotton blue solution slides and observed and photographed using a compound microscope (Nikon ECLIPSE Ni) with a digital camera (Canon EOS 600D) fitted on to the top of the microscope. These important fungal structures were measured with the Tarosoft (R) Image Frame Work program and the images used were processed with Adobe Photoshop CS3 Extended v. 10.0 (Adobe^®^, San Jose, CA, United States).

### DNA Extraction, PCR Amplification, and Sequencing

The total DNA was extracted from stromal tissue of specimens HKAS 102552, HKAS 102553, HKAS 102484 and from fresh mycelium of KUMCC 20-0001 (ex-type culture of isolate HKAS 102546) using DNA extraction kit (Omega Fungus Genomic DNA Extraction Kit, China), following the protocol of the manufacturer. The obtained DNA was stored at −20°C in a refrigerator. The PCR amplification was performed in 25 μL volumes consisting 12.5 μL PCR mixture (2 × Taq PCR Master Mix, red dye) which contains Taq DNA polymerase, dNTPs, MgCl_2_, a reaction buffer, a PCR reaction enhancer, an optimizer and stabilizer, 8.5 μL of twice-sterilized water, 1 μL of each primer and 2 μL of 30 μg/μl DNA template. The internal transcribed spacer (ITS1-5.8S-ITS2, ITS), large subunit ribosomal RNA (LSU rRNA), small subunit ribosomal RNA (SSU rRNA), translation elongation factor 1-alpha gene (TEF1-α) and RNA polymerase II largest subunit (RPB1) and RNA polymerase II second largest subunit (RPB2) were amplified with the primers and procedures mentioned in Table 1. The PCR products were sent to Tsingke company, Yunnan Province, China, for sequencing the above genes. The generated sequences were submitted to GenBank, and the accession numbers have been shown in Table 2.

**TABLE 1:** Gene and primers used in the phylogenetic analyses.

Gene (reference)	Primer	Sequences	PCR condition
LSU (Vilgalys and Hester, 1990)	LROR	ACCCGCTGAACTTAAGC	(1) Initialization at for 3 min at 94°C. (2) 40 cycles of denaturation at 94°C for 45 s, annealing at 56°C for 50 s, and extension at 72°C for 1 min. (3) final elongation at 72°C for 10 min and (4) storage at 4°C.
	LR5	TCCTGAGGGAAACTTCG	
SSU (White et al., 1990)	NS1	GTAGTCATATGCTTGTCTC	
	NS4	CTTCCGTCAATTCCTTTAAG	
ITS (White et al., 1990)	ITS4	TCCTCCGCTTATTGATATGC	
	ITS5	GGAAGTAAAAGTCGTAACAAGG	
RPB1 (Castlebury et al., 2004)	CRPB1Af	CAYCCWGGYTTYATCAAGAA	(1) Initialization at 94°C for 2 min, (2) 10 cycles of denaturation at 94°C for 30 s, annealing at 64°C for 1 min, and extension at 72°C for 1 min, (3) followed by 35 cycles of denaturation at 94°C for 30 s, annealing at 54°C for 1 min, and extension at 72°C for 1 min and (4) final elongation at 72°C for 3 min. (5) storage at 4°C.
	CRPB1Cr	CCNGCDATNTCRTTRTCCATRTA	
TEF1-α (Rehner and Buckley, 2005)	983F	GCYCCYGGHCAYCGTGAYTTYAT	
	2218R	ATGACACCRACRGCRACRGTYTG	
RPB2 (Liu et al., 1999; Sung et al., 2007b)	RPB2-5F RPB2-7cR	GAYGAYMGWGATCAYTTYGG CCCATRGCTTGTYYRCCCAT	(1) Initialization at 95°C for 3 min. (2) 40 cycles of denaturation at 95°C for 1 min, annealing at 52°C for 2 min, and extension at 72°C for 90 s. (3) final elongation at 72°C for 10 min and (4) storage at 4°C.

**TABLE 2:** GenBank accession numbers of the taxa used in the phylogenetic analyses.

Species	Specimen number	SSU	LSU	TEF1-α	RPB1	RPB2	ITS	References
*Ophiocordyceps highlandensis*	HKAS 83206	KM581282	**–**	**–**	KM581274	KM581278	**–**	Yang et al., 2015
*Ophiocordyceps highlandensis*	HKAS 83207	KM581284	**–**	**–**	KM581276	KM581280	**–**	Yang et al., 2015
*Ophiocordyceps konnoana*	EFCC 7295	EF468958	**–**	**–**	EF468862	EF468915	**–**	Araújo et al., 2018
*Ophiocordyceps konnoana*	EFCC 7315	EF468959	**–**	EF468753	EF468861	EF468916	**–**	Araújo et al., 2018
*Ophiocordyceps melolonthae*	OSC 110993	DQ522548	DQ518762	DQ522331	DQ522376	**–**	**–**	Sung et al., 2007a
*Ophiocordyceps melolonthae*	Ophgrc679	**–**	KC610768	KC610744	KF658666	**–**	**–**	Araújo et al., 2018
*Ophiocordyceps nigrella*	EFCC 9247	EF468963	EF468818	EF468758	EF468866	EF468920	**–**	Araújo et al., 2018
*Ophiocordyceps ravenelii*	OSC 110995	DQ522550	DQ518764	DQ522334	DQ522379	DQ522430	**–**	Araújo et al., 2018
*Ophiocordyceps ravenelii*	OSC 151914	KJ878932	**–**	KJ878978	KJ879012	KJ878950	**–**	Araújo et al., 2018
*Ophiocordyceps superficialis*	MICH 36253	EF468983	**–**	**–**	EF468883	**–**	**–**	Sung et al., 2007a
*Ophiocordyceps variabilis*	ARSEF 5365	DQ522555	DQ518769	DQ522340	DQ522386	DQ522437	**–**	Araújo et al., 2018
*Ophiocordyceps variabilis*	OSC 111003	EF468985	EF468839	EF468779	EF468885	EF468933	**–**	Araújo et al., 2018
***Paraisaria alba***	**HKAS 102484**	**MN943843**	**MN943839**	**MN929085**	**MN929078**	**MN929082**	**MN947219**	**This study**
*Paraisaria amazonica*	HUA 186143	KJ917562	KJ917571	KM411989	KP212902	KM411982	**–**	Ban et al., 2015
*Paraisaria amazonica*	HUA 186113	KJ917566	KJ917572	**–**	KP212903	KM411980	**–**	Ban et al., 2015
***Paraisaria arcta***	**HKAS 102553**	**MN943845**	**MN943841**	**MN929087**	**MN929080**	**–**	**MN947221**	**This study**
***Paraisaria arcta***	**HKAS 102552**	**MN943844**	**MN943840**	**MN929086**	**MN929079**	**MN929083**	**MN947220**	**This study**
*Paraisaria blattarioides*	HUA186093	KJ917559	KJ917570	KM411992	KP212910	**–**	**–**	Ban et al., 2015
*Paraisaria blattarioides*	HUA 186108	KJ917558	KJ917569	**–**	KP212912	KM411984	**–**	Ban et al., 2015
*Paraisaria coenomyiae*	NBRC 106964	AB968385	AB968413	AB968571	**–**	AB968533	AB968397	Ban et al., 2015
*Paraisaria coenomyiae*	NBRC 108993	AB968384	AB968412	AB968570	**–**	AB968532	AB968396	Ban et al., 2015
*Paraisaria gracilioides*	HUA 186095	KJ917556	**–**	KM411994	KP212914	**–**	**–**	Li et al., 2004
*Paraisaria gracilioides*	HUA 186092	KJ917555	KJ130992	**–**	KP212915	**–**	**–**	Mongkolsamrit et al., 2019
*Paraisaria gracilis*	EFCC 3101	EF468955	EF468810	EF468750	EF468858	EF468913	**–**	Araújo et al., 2018
*Paraisaria gracilis*	EFCC 8572	EF468956	EF468811	EF468751	EF468859	EF468912	**–**	Araújo et al., 2018
*Paraisaria heteropoda*	OSC 106404	AY489690	AY489722	AY489617	AY489651	**–**	**–**	Araújo et al., 2018
*Paraisaria heteropoda*	EFCC 10125	EF468957	EF468812	EF468752	EF468860	EF468914	JN049852	Araújo et al., 2018
*Paraisaria orthopterorum*	BBC 88305	**–**	MK332583	MK214080	MK214084	**–**	MH754742	Mongkolsamrit et al., 2019
*Paraisaria orthopterorum*	TBRC 9710	**–**	MK332582	MK214081	MK214085	**–**	MH754743	Mongkolsamrit et al., 2019
*Paraisaria phuwiangensis*	BBH 43491	**–**	MK192058	**–**	MH211351	**–**	MH188542	Mongkolsamrit et al., 2019
*Paraisaria phuwiangensis*	TBRC 9709	**–**	MK192057	MK214082	MK214086	**–**	MK192015	Mongkolsamrit et al., 2019
*Paraisaria phuwiangensis*	BBH 43492	**–**	MH201169	MH211355	MH211352	**–**	MH188541	Mongkolsamrit et al., 2019
***Paraisaria rosea***	**HKAS 102546**	**MN943846**	**MN943842**	**MN929088**	**MN929081**	**MN929084**	**MN947222**	**This study**
*Paraisaria tettigonia*	GZUH CS14062709	KT345955	**–**	KT375440	KT375441	**–**	KT345954	Wen et al., 2016
*Paraisaria yodhathaii*	BBH 43163	**–**	MK332584	MH211353	MH211349	**–**	MH188539	Mongkolsamrit et al., 2019
*Paraisaria yodhathaii*	TBRC 8502	**–**	MH201168	MH211354	MH211350	**–**	MH188540	Mongkolsamrit et al., 2019
*Polycephalomyces formosus*	ARSEF 1424	KF049615	KF049634	KF049689	KF049651	KF049671	KF049661	Xiao et al., 2018
*Polycephalomyces nipponicus*	BCC 2325	KF049622	KF049640	KF049696	KF049655	KF049677	KF049665	Xiao et al., 2018
*Polycephalomyces ramosopulvinatus*	EFCC 5566		KF049627	KF049682	KF049645	**–**	KF049658	Xiao et al., 2018
*Polycephalomyces ramosus*	MFLU 18-0162	MK863043	MK863050	**–**	**–**	**–**	MK863250	Xiao et al., 2018
*Purpureocillium lilacinum*	CBS 284.36	**–**	**–**	EF468792	EF468898	**–**	AY624189	Mongkolsamrit et al., 2019
*Purpureocillium lilacinum*	CBS 431.87	**–**	EF468844	EF468791	EF468897	**–**	AY624188	Mongkolsamrit et al., 2019
*Purpureocillium takamizusanensis*	NHJ 3497	EU369096	EU369033	EU369014	EU369053	EU369074	**–**	Sung et al., 2007a
*Tolypocladium capitatum*	NBRC 106327	JN941737	JN941404	**–**	JN992471	**–**	JN943317	Mongkolsamrit et al., 2019
*Tolypocladium inflatum*	CBS 567.84	**–**	MH873477	**–**	**–**	**–**	MH861779	Mongkolsamrit et al., 2019
*Tolypocladium inflatum*	CBS 127142	**–**	MH875875	**–**	**–**	**–**	MH864435	Mongkolsamrit et al., 2019
*Tolypocladium japonicum*	OSC 110991	DQ522547	DQ518761	DQ522330	DQ522375	DQ522428	JN049824	Mongkolsamrit et al., 2019
*Tolypocladium ophioglossoides*	NBRC 106331	JN941733	JN941408	**–**	JN992467	**–**	JN943320	Mongkolsamrit et al., 2019
*Drechmeria gunnii*	OSC 76404	AF339572	AF339522	AY489616	AY489650	DQ522426	JN049822	Mongkolsamrit et al., 2019
*Drechmeria balanoides*	CBS 250.82	AF339588	AF339539	DQ522342	DQ522388	DQ522442	MH861495	Mongkolsamrit et al., 2019
*Harposporium anguillulae*	ARSEF 5407	**–**	AY636080	**–**	**–**	**–**	**–**	Mongkolsamrit et al., 2019
*Harposporium anguillulae*	ARSEF 5593	**–**	AY636081	**–**	**–**	**–**	**–**	Mongkolsamrit et al., 2019
*Harposporium helicoides*	ARSEF 5354	AF339577	AF339527	**–**	**–**	**–**	**–**	Mongkolsamrit et al., 2019
*Perennicordyceps prolifica*	NBRC 100744	JN941709	JN941432	**–**	JN992443	**–**	**–**	Mongkolsamrit et al., 2019
*Perennicordyceps prolifica*	NBRC 101750	JN941708	JN941433	**–**	JN992442	**–**	JN943340	Ban et al., 2009
*Perennicordyceps prolifica*	NBRC 103838	JN941707	JN941434	**–**	JN992441	**–**	JN943339	Ban et al., 2009
*Perennicordyceps cuboidea*	NBRC 100941	**–**	AB378646	**–**	**–**	**–**	AB378666	Ban et al., 2009
*Perennicordyceps cuboidea*	NBRC 101742	**–**	AB378648	**–**	**–**	**–**	AB378667	Ban et al., 2009
*Cordyceps militaris*	OSC 93623	AY184977	AY184966	DQ522332	DQ522377	**–**	JN049825	Kepler et al., 2013
*Cordyceps kyusyuensis*	EFCC 5886	EF468960	EF468813	EF468754	EF468863	EF468917	**–**	Kepler et al., 2013

*The new species generated in this study are in black bold.*

### Sequence Alignment and Phylogenetic Analyses

The generated sequences were assembled with Sequencing Project Management (SeqMan) (Clewley, 1995). The sequences for the combined alignment were selected based on the blast results of LSU, SSU, ITS, TEF, RPB1, and RPB2 as well as the recent references listed in Table 2. The individual gene alignment was aligned in MAFFT v. 7 web server^1^ (Kuraku et al., 2013; Katoh et al., 2019). The alignments of each locus were improved by manually removing uninformative gaps and ambiguous regions using BioEdit v. 7.0.9.1 (Hall, 1999) and were concatenated in Sequence Matrix v. 1.7.8 (Vaidya et al., 2011). The final combined alignment was converted to a NEXUS file (.nex) with ClustalX2 v. 1.83 (Thompson et al., 1997) and was used for Bayesian inference (BI) analysis and Maximum parsimony analysis (MP). The optimum nucleotide substitution model of each gene was selected by MrModeltest v.2.3 (Nylaner, 2004) using the Akaike information criterion (AIC) method and was applied to Bayesian inference (BI) analysis that was performed using MrBayes on XSEDE (2.2.7a) (Ronquist and Huelsenbeck, 2003) on CIPRES Science Gateway^2^. The Bayesian posterior probability (BYPP) was estimated by the Markov Chain Monte Carlo (MCMC) technique. Six simultaneous Markov Chains were run for 2,000,000 generations with sampling every 1,000 generation. The first 25% of sampled trees were discarded during the burn-in period. Maximum likelihood analysis was carried out using RAxML-HPC2 on XSEDE (8.2.10) in CIPRES Science Gateway V. 3.3 (Miller et al., 2010), with default algorithm and bootstrap iterations were set to 1,000 and substitution model was set to GTRGAMMA + I. Maximum parsimony analysis was implemented in PAUP v. 4.0b10 (Swofford, 2002) through heuristic search with 1,000 random replicates of stepwise addition and tree-bisection-reconnection (TBR) of branch-swapping algorithm. Gaps were treated as missing data and max trees was set to 1,000. Branches collapsed when minimum branch length was zero. The consistency index (CI), retention index (RI), rescaled consistency index (RC) and homoplasy index (HI) were calculated for the maximum parsimony tree. For the delimitation of new species based on nucleotide comparison, we follow the suggestion of Jeewon and Hyde (2016).

The tree topologies were visualized in FigTree v1.4.0 (Rambaut, 2006) and edited in Microsoft power point (2016) and Adobe Photoshop CS3 Extended v. 10.0 (Adobe^®^, San Jose, CA, United States). The final alignment and trees were submitted to TreeBASE with submission number 25664^3^.

## Results

### Phylogenetic Analyses

Phylogenetic analyses were constructed with combined LSU, SSU, TEF1-α, RPB1, RPB2, and ITS sequences data of 58 representative taxa in Ophiocordycipitaceae. Trees were rooted to *Cordyceps militaris* (OSC 93623) and *C. kyusyuensis* (EFCC5886) in Cordycipitaceae. The alignment contains 5239 characters, including gaps (LSU: 918, SSU: 1027, TEF1-α: 906, RPB1: 664, RPB2: 1024, ITS: 700). Parsimony analysis of this dataset produced the 20 most parsimonious trees of 4833 steps in length, of which 3436 characters were constant, 380 variable characters parsimony-uninformative and 1423 characters parsimony-informative. The first parsimonious tree was represented as the best tree, with CI = 0.549, RI = 0.777, RC = 0.426 and HI = 0.451. The RAxML analysis of the combined dataset yielded a best scoring tree with a final ML optimization likelihood value of −30766.070218. The matrix had 2305 distinct alignment patterns, with 41.28% undetermined characters or gaps. Estimated base frequencies were as follows: A = 0.236752, C = 0.277080, G = 0.283017, T = 0.203151; substitution rates AC = 1.485223, AG = 3.851975, AT = 0.915108, CG = 1.456245, CT = 6.890167, GT = 1.000000; gamma distribution shape parameter α = 0.465094.

**FIGURE 1:**
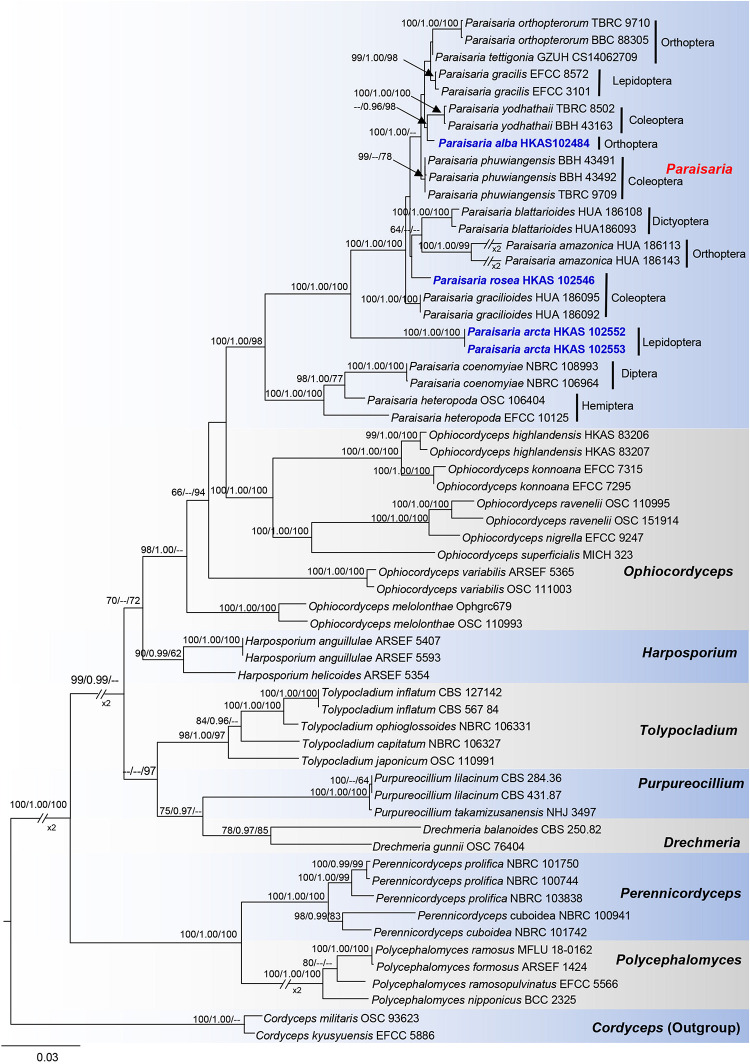
Phylogram generated from maximum likelihood analysis based on combined LSU, SSU, TEF1-α, RPB1, RPB2, and ITS sequence data. Bootstrap values for BI equal to or higher than 95%, ML and MP equal to or greater than 60% are placed on the notes. The newly generated sequences are indicated in blue bold. The host order of *Paraisaria* species and the generic names are labeled in the right side.

In the phylogenetic analyses (Figure 1), eight genera are included in Ophiocordycipitaceae labeled on the tree. With the exception of *Ophiocordyceps*, the other remaining genera are monophyletic and individually they received strong statistical support. The three novel entomopathogenic fungi grouped with the taxa in *Paraisaria* with significant statistical support (1.00 PP/100% ML/98% MP). *Paraisaria alba* (HKAS 102484) constitutes a sister phylogenetic affiliation to *P. yodhathaii* with 0.96 PP/98% MP statistical support. *Paraisaria rosea* (HKAS 102546) is closely related to *P. amazonica* and *P. blattarioides*, but this is statistically not supported in all three formats. Two strains of *P. arcta* grouped as an intermediate clade with close phylogenetic connection to *P*. *coenomyiae*, *P*. *gracilioides*, and *P*. *heteropoda*.

### Taxonomy

#### *Paraisaria alba* D. P. Wei and K. D. Hyde, sp. nov. Figure 2

Etymology: *alba* refers to the white fertile head.

**FIGURE 2:**
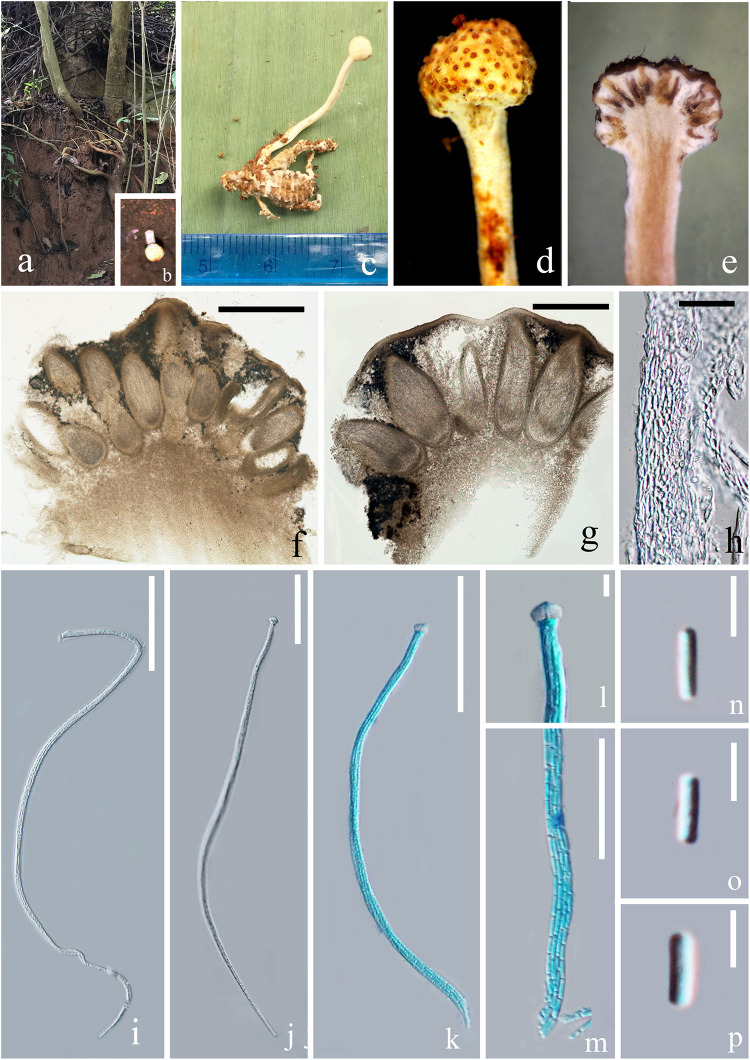
*Paraisaria alba* (HKAS 102484, holotype). **(a)** Habitat. **(b)** Host imbedded into the soil with the stroma emerging from the ground. **(c)** Stroma arising from host. **(d)** Fertile head. **(e)** Longitudinal section through fertile head. **(f,g)** Perithecia. **(h)** Peridium. **(i–k)** Asci. **(l)** Ascus cap. **(m)** Part of the asci. **(n–p)** Secondary ascospores. Scale bars: **(f)** 500 μm, **(g)** 200 μm, **(h)** 20 μm, **(i–k)** 50 μm, **(m)** 30 μm, **(l,n–p)** 5 μm. (k**–**m mounted in cotton blue reagent.).

MycoBank number: MB 833999

Facesoffungi number: FoF 07239

*Parasitic* on an adult cricket (Orthoptera). **Sexual morph:** *Stroma* up to 26 mm in tall, single, unbranched, growing from the flank of the host. *Fertile head* 3.5 mm in diam., globose, white when fresh, yellow brown when dry. *Stipe* 22.5 × 1.2 mm, slightly flexuous, fleshy, white, glossy, not hollow. *Perithecia* 200–500 × 100–220 ($\bar{x}$ = 325 × 145, *n* = 20) μm, immersed, ovoid. *Asci* 160–250 × 2.5–5 ($\bar{x}$ = 200 × 3.5, *n* = 10) μm, unitunicate, hyaline, narrow cylindrical, attenuated toward the base, with thickened cap. *Peridium* 10–40 ($\bar{x}$ = 20, *n* = 30) μm in thick, comprising hyaline, thick-walled cell of *textura angularis*. *Apical cap* 4.6–7.4 × 3.2–4.9 ($\bar{x}$ = 6 × 3.8, *n* = 30) μm, with a narrow tunnel throughout the center. *Ascospores* filiform, equal to the asci in length, when mature, breaking into numerous secondary ascospores. *Secondary ascospores* 3–5 × 0.5–1.5 ($\bar{x}$ = 4 × 1, *n* = 30) μm, cylindrical, hyaline, smooth, one-celled, straight, with truncated ends.

##### Material examined

**Thailand**, Krabi, Plai Phraya (N: 8°24′410′′, E: 98°45′34′′). On an adult cricket, 20 December 2018, *Deping Wei*, 211-1(HKAS 102484– holotype). We tried to culture *P. alba* by transferring a small piece of inner stroma tissue into a PDA medium using a sterilized needle, but growth was not observed.

##### Notes

The multigene phylogenetic analysis showed that *P. alba* groups with *P. yodhathaii* with fairly good statistical support (0.96 PP/98% MP, Figure 1). This relationship is, however, not supported by the ML analysis. *Paraisaria alba* differs from *P. yodhathaii* in having solitary stroma, a white fertile head, and smaller perithecia, asci and secondary ascospores, whereas *P. yodhathaii* has paired stromata, grayish yellow fertile head, larger perithecia and larger asci and secondary ascospores (Table 3). The comparison of the nucleotide sequences between *P. alba* and *P. yodhathaii* show 10 (including 6 gaps) out of 410 bp (2.4%), 6 out of 746 bp (0.8%), 5 out of 881 bp (0.56%) and 8 out of 534 bp differences (1.5%) in ITS, LSU, TEF1-α and RPB1 sequences, respectively. SSU and RPB2 sequences data of *P. yodhathaii* are not available in GenBank. Henceforth, we describe our collection as a new species in *Paraisaria* according to the guidelines of Jeewon and Hyde (2016).

**TABLE 3:** Synopsis of *Paraisaria* species discussed in this study.

Species	Host	Distribution	Stroma (mm)	Fertile part (mm)	Perithecia (μm)	Asci (μm)	Part-ascospores (μm)	Asexual morphs
***P. alba***	Adult cricket (Orthoptera)	Thailand: Krabi Province	Solitary, 26 long	Globose, white, 3.5 in diam.	Ovoid, 200–500 × 100–220	160–250 × 2.5–5	3–5 × 0.5–1.5	Absent
*P. amazonica*^*a,d,h*^	Adult or imago of Acrididae (Orthoptera)	Colombia and Ecuador	Gregarious, 20–45 long	Subglobose to spherical, reddish brown, 2.5–5.5	Ovoid-ellipsoidal, 760–1100 × 220–400	325–450 × 5	9–17 × 0.5–2	Absent
***P. arcta***	Larva of Lepidoptera	China: Guizhou Province	Solitary, 16 long	Subglobose with constriction at center, white, 2 × 3	Ampulliform to ellipsoidal, 230–530 × 70–180	100–180 × 2–4	2.6– 4.2 × 0.5–1.3	Absent
*P. blattarioides*^*c,h*^	Adult of Blattaria (Dictyoptera)	Belize, Colombia and Ecuador	Gregarious, 14–20 long	Ovoid, subglobose, chestnut brown, 2–3 × 1.5–2.5	Ellipsoidal, 650–800 × 220–300	180–250(–300) × 4–5	6–16 × 1.5	Absent
*P. coenomyiae*^*b*^	Larva of *Coenomyia* (Diptera)	Japan	Solitary, 30–35 long	Ovoid, subglobose, chestnut brown, 8 × 10	Lanceolate, 700–750 × 200–220	500–750 × 7.8–8.0	8–15 × 1.8–2.5	Absent
*P. gracilioides*^*b,e,h*^	Larva of *Elateridae* (Coleoptera)	Bolivia, China, Colombia, Japan and Mexico	Usually solitary, 20–90 long	Spherical, pale rufous, 4–5.5	Ellipsoidal to naviform, 680–900 × 200–280	450–700 × 5–6.5	7–12 × 1–2	Present
*P. gracilis*^*d,g,h*^	Larva of *Hepialidae* (Lepidoptera)	Africa, America, Asia, Europe, and Oceania	Usually solitary, 40– 90 long	Globose to ellipsoidal, red ochreous to pale orange, 4–9 × 4–7	Elongate to oviform, (320–)560–840 × 200–360	(200–)400–528 × 5–8	5–9 × 1.5–2	Present
*P. heteropoda*^*e*^	Nymph of Cicadidae (*Hemiptera*)	Australia, Japan	Solitary, 120 long	Ovoid, cinnamon buff, 7–9 × 6–7	Ampulliform, 610–660 × 210	250–300 × 5.2–7	6–7.7 × 0.9–1	Present
*P. myrmicarum*	*Myrmica rubra* (Hymenoptera)	United Kingdom	–	–	–	–	–	Present
*P. orthopterorum*^*f*^	Nymph of Orthoptera	Thailand: Trat Province	Solitary, 10–45 long	Globose, gray orange, 2–4 × 3	Obclavate, 520–650 × 150–250	400 × 5	5–10 × 1–1.5	Present
*P. phuwiangensis*^*f*^	Larva of Elateridae (Coleoptera)	Thailand: Khon Kaen Province	Solitary, 30–50 long	Globose to subglobose, light brown, 4–8 × 4–7	Obpyriform, 800–1200 × 300–380	500 × 3–5	5–10 × 1–2	Present
***P. rosea***	Larva of Coleoptera	China: Yunnan Province	Solitary, 14.5 long	Subglobose, pale pink, 4.5 × 4	Ampulliform, 500–900 × 150–350	230–390 × 3.5–6	4–11 × 1.5–2.5	Present
*P. tettigonia*^*f*^	Adult of *Tettigonia* (Orthoptera)	China: Guizhou Province	Paired, 32.5–37.5 long	Globose, white, 2–2.5	Elongated to ampulliform, 520–680 × 205–275	530–615 × 6.5–9.3	6.7–9.4 × 1.5–2.3	Absent
*P. yodhathaii*^*f*^	Larva of *Elateridae* (Coleoptera)	Thailand: Khon Kaen Province	Gregarious, 20–35 long	Globose to subglobose, grayish yellow, 2–4 × 2–5	Obclavate, 650–800 × 160–250	480 × 5–6	5–10 × 1–2	Present

*The new species generated in this study are in bold.**^*a*^**Ban et al., 2009; **^*b*^**Ban et al., 2015; **^*c*^**Hennings, 1904; **^*d*^**Kobayasi, 1941; **^*e*^**Mains, 1940; **^*f*^**Mongkolsamrit et al., 2019; **^*g*^**Samson and Brady, 1983; **^*h*^**Sanjuan et al., 2015; **^*i*^**Wen et al., 2016.*

#### *Paraisaria arcta* D. P. Wei and K. D. Hyde, sp. nov. Figure 3

Etymology: ***arcta*** refers to the constricted fertile head.

**FIGURE 3:**
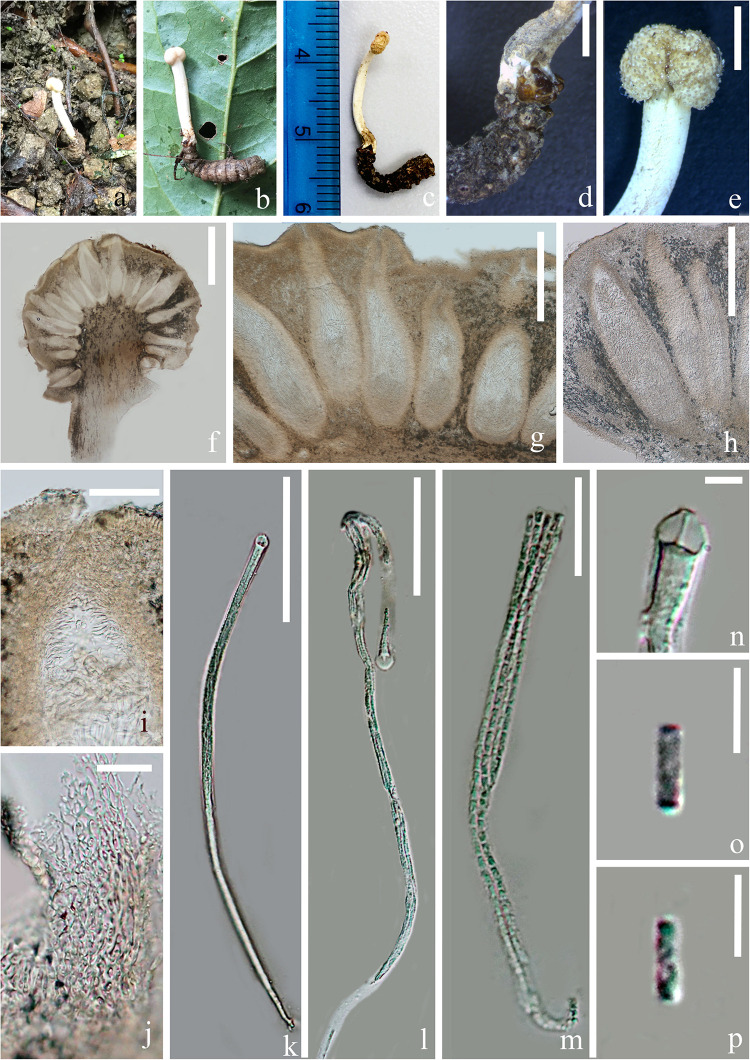
*Paraisaria arcta* (HKAS 102553, holotype). **(a)** Substrate. **(b–d)** Stromata emerging from host mouth. **(e)** Fertile head. **(f–h)** Perithecia. **(i)** Ostiole. **(j)** Peridium. **(k,l)** Asci. **(m)** Parts of ascus. **(n)** Ascus cap. **(o,p)** Secondary ascospores. Scale bars: **(d,e)** 2000 μm, **(f)** 500 μm, **(g,h)** 200 μm, **(i,k)** 50 μm, **(j,m)** 15 μm, **(l)** 30 μm, (**n–p) 3** μm.

MycoBank number: MB 834000

Facesoffungi number: FoF 07240

*Parasitic* on larva of Lepidopteran larva. **Sexual morph:** *Stroma* 16 mm long, single, arising from the mouth of host larva. *Fertile head* 2 mm long, 3 mm wide, white, nearly globose, constricted at the center, with sticky and crystal-like substance on the surface. *Stipe* 14 mm long, 2 mm wide, straight, fleshy, white, glossy. *Perithecia* 230–530 × 70–180 ($\bar{x}$ = 387 × 113, *n* = 20) μm, completely immersed, ampulliform to ellipsoid. *Peridium* 14–20 ($\bar{x}$ = 17, *n* = 30) μm wide, composed of hyaline, thick-walled, smooth-walled cells of *textura angularis*. *Asci* 100–180 × 2–4 μm ($\bar{x}$ = 137 × 2.9, *n* = 15), unitunicate, hyaline, narrow cylindrical, tapering toward the base, 8-spored, with thickened cap. *Apical cap* 3.5–4.5 × 2–3.6 μm thick ($\bar{x}$ = 4 × 2.8, *n* = 20), with a narrow tunnel throughout the center. *Ascospores* hyaline, narrow filiform, equal to the asci in length, when mature, breaking into numerous secondary ascospores. *Secondary ascospores* 2.6–4.2 × 0.5–1.3 μm ($\bar{x}$ = 3.3 × 0.9, *n* = 60), cylindrical, with truncated ends, hyaline, smooth, one-celled, straight.

##### Material examined

**China,** Guizhou Province, Qianxinan Buyei and Miao Autonomous Prefecture, Ceheng County, Gaofeng Villige (N: 24°57′33′′, E: 105°50′1′′), on dead larva of *Lepidoptera* sp., 6 August 2018, *Deping Wei*, GFC604 (HKAS 102553–holotype); GFC603 (HKAS 102552 – paratype). The culturing of *P. arcta* was tried by transferring a mass of mycelium found inside body of the larva host to a PDA medium using a sterilized needle. However, mycelium growth was not observed.

##### Notes

*Paraisaria arcta* resembles *P. alba* found in Krabi Province, Thailand and *P. tettigonia* discovered in Guizhou Province, China in having white fertile heads but differs from *P. alba* in its associated host and number of stromata are distinct from *P. tettigonia* (Wen et al., 2016). *Paraisaria arcta* can also be distinguished from the other species in *Paraisaria* by the color and shape of its fertile head. A conspicuous ravine throughout the center of the fertile head is present in *P. arcta*, which is lacking in the other species in this genus. The detailed comparisons are shown in Table 3. Multigene phylogenetic analysis showed *P. arcta* constitutes a distant clade from other species in *Paraisaria*, with strong statistical support (100% ML, 100% MP, 1.00 PP, Figure 1). Herein, we introduce this collection as a new species of *Paraisaria.*

#### *Paraisaria rosea* D. P. Wei and K. D. Hyde, sp. nov. Figures 4, 5

Etymology: *rosea* refers to its pink fertile head.

**FIGURE 4:**
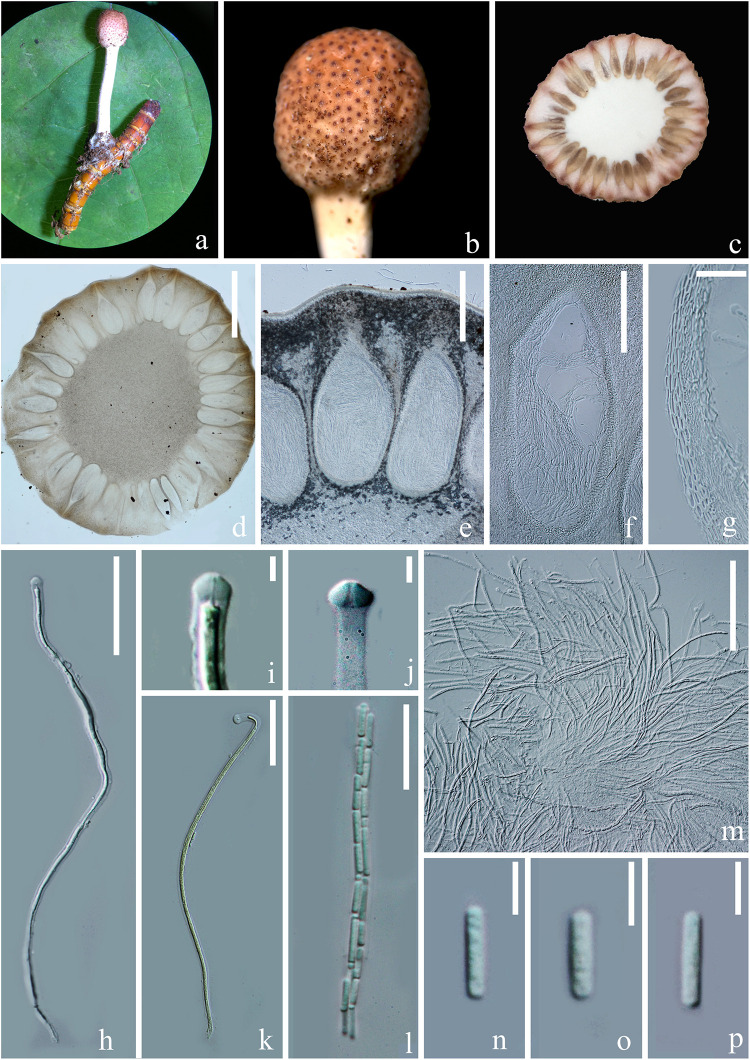
Sexual morph of *Paraisaria rosea* (HKAS 102546, Holotype). **(a)** Stroma emerging from host. **(b)** Fertile head. **(c)** Transverse section of the fertile head. **(d–f)** Perithecia. **(g)** Peridium. **(h,k,m)** Asci. **(i,j)** Asci cap. **(l)** Part of ascus. **(n–p)** Secondary ascospores. Scale bars: **(d)** 1000 μm, **(e)** 300 μm, **(f)** 200 μm, **(g,m)** 30 μm, **(h,k)** 50 μm, **(l)** 20 μm, **(i,j, n–p)** 5 μm. (**k** mounted in Melzer’s reagent).

**FIGURE 5:**
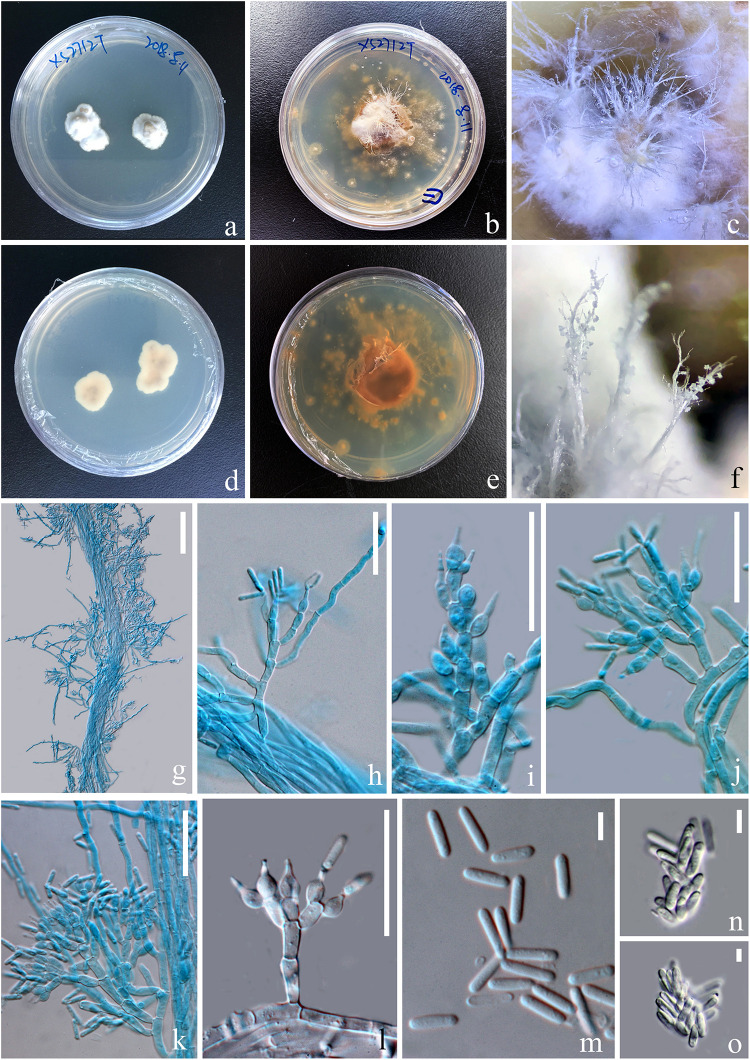
Asexual morph of *Paraisaria rosea* (KUMCC 20-0001, ex-type). **(a,d)** Upper and lower views of cultures on PDA after 50 days. **(b,e)** Upper and lower views of cultures on PDA after 16 months incubation in dark environments. **(c,f)** Enlargement of aerial synnemata produced on culture. **(g)** Synnema bearing conidiophores. **(h–l)** Phialides. **(m)** Conidia. **(n,o)** Irregularly aggregated conidia. Scale bars: **(g)** 100 μm, **(h–l)** 30 μm, **(m–o)** 5 μm. (**h–k,m** mounted in cotton blue reagent.).

MycoBank number: MB834001

Facesoffungi number: FoF 07241

*Parasitic* on a larva of Coleoptera. Host buried in the soil, with the stroma erumpent from the ground. **Sexual morph:** *Stroma* up to 14.5 mm long, laterally emerging from the middle part of the larva body, simple, erect. *Fertile head* 4.5 × 4 mm, subglobose, pale pink at top and paler toward the base when fresh, pale yellow-brown when dry. *Stipe* 10 × 1.5 mm, white, straight, unbranched, glossy, cylindrical, inside not hollow. *Perithecia* 500–900 × 150–350 ($\bar{x}$ = 762 × 256, *n* = 30) μm, completely immersed, ampulliform, ostiolate. *Peridium* 9–15 ($\bar{x}$ = 12, *n* = 30) μm wide, composed of hyaline, thick-walled cells of *textura angularis* to *textura globulosa* to *textura prismatica*. *Asci* 230–390 × 3.5–6 ($\bar{x}$ = 280 × 5, *n* = 15) μm, hyaline, cylindrical, unitunicate, eight-spored, possessing a prominent apical cap. *Apical cap* 5–7 × 2–6 ($\bar{x}$ = 6 × 4, *n* = 20) μm, with a conspicuous tunnel throughout the center. *Ascospores* filiform, hyaline, breaking into secondary ascospores when mature. *Secondary ascospores* 4–11 × 1.5–2.5 ($\bar{x}$ = 7.5 × 2, *n* = 30) μm, hyaline, cylindrical with truncate ends, smooth-walled, aseptate. **Asexual morph:** Hyphomycetous. *Synnemata* producing from the center of culture after 16 months incubation in dark environment, composed of loose, septate hyphae, white, filamentous, aerial, straight, branched, fasciculate, bearing shining droplets and conidiophores. *Mycelium* 2.4–3.7 ($\bar{x}$ = 3, *n* = 10) μm in wide, septate, hyaline, smooth-walled. *Conidiophores* 33–48 ($\bar{x}$ = 41, *n* = 10) μm in height, irregularly differentiate from the synnemata, sparse, gregarious, branched. *Phialides* 5.8–11.5 × 3–5.5 ($\bar{x}$ = 8.6 × 4, *n* = 30) μm, ampulliform, 1-necked, hyaline, aseptate, enteroblastic, phialidic, monophialidic. *Conidia* 8–12 × 2–2.6 ($\bar{x}$ = 9.8 × 2.3, *n* = 50) μm, hyaline, cylindrical, smooth-walled, aseptate, with round ends.

##### Culture characteristics

Culture was made from mycelium inside body of the host larva, slowly growing on PDA, reaching 1.3 cm in diam after incubated at room temperature (25°C) for 50 days, convex, dense, with undulate edges, smooth surface become filamentous after forming aerial synnemata. The shooting conidia land on the surrounding culture and develop new colonies.

##### Material examined

**China**, Yunnan Province, Kunming, Western hill Park (N: 24°57′28′′, E: 102°38′17′′), on larva of *Coleoptera* sp. buried in soil, 27 July 2018, *Deping Wei*, XS2712 (HKAS 102546 – Holotype); (KUMCC 20-0001 – ex-type living culture).

##### Notes

*Paraisaria rosea* is closely related to *P. amazonica* and *P. blattarioides*, without any statistical support (Figure 1). However, *P. rosea* can be distinguished from these related species based on the number of stromata, the color of the fertile head and the size of asci and secondary ascospores (Table 3). The ITS sequence of *P. amazonica* and *P. blattarioides* are not available in GenBank database; the nucleotide differences in the TEF1-α, RPB1 and RPB2 region between *P. rosea* and the two above species are greater than 1.5% (Table 4). Thereby, we introduced *P. rosea* as a new species in this genus based on the distinctive morphology and molecular support.

**TABLE 4:** The comparison of nucleotide sequences between *Paraisaria rosea* and two close species.

Species	TEF1-α (bp)	RPB1 (bp)	RPB2 (bp)
*Paraisaria amazonica*	4.4% (38/862)	5.7% (37/642)	4.3% (31/711)
*Paraisaria blattarioides*	1.6% (14/862)	2.5% (16/629)	–

## Discussion

The sexual morph of *Paraisaria* species phenotypically share an erect or slightly flexuous, cylindrical, colorless, fleshy stipe that terminates in a subglobose to globose fertile head and completely immersed perithecia. Asci are cylindrical with a thickened apical cap. Ascospores are hyaline, multi-septate and usually break into numerous cylindrical, truncated fragments at maturity. However, they can be distinguished according to their associated host, the number of stroma and the color of the fertile head. Species in this genus usually infect several stages of insects, such as larvae of Coleoptera, Diptera, and Lepidoptera; nymphs of Hemiptera and Orthoptera; or adults of Dictyoptera, Hymenoptera (ant) and Orthoptera (Evans et al., 2010; Sanjuan et al., 2015; Mongkolsamrit et al., 2019). According to the number of stromata, species of *Paraisaria* can be divided into three groups: solitary stroma, paired stromata and multiple stromata (see the key below). The shape of their fertile head features little variation, though differing in color, ranging from white, pale pink, pale rufous, red ochreous to pale orange, chestnut, cinnamon buff, grayish, reddish brown to dark brown (see Table 3).

The asexual morphs of this genus are known in eight species, viz. *P. myrmicarum* (Evans et al., 2010), *P. gracilis* (Samson and Brady, 1983), *P. gracilioides* (Li et al., 2004), *P. rosea* (this study), *P. heteropoda*, *P. orthopterorum*, *P. phuwiangensis*, and *P. yodhathaii* (Mongkolsamrit et al., 2019). Their conidiophores are irregularly branched and generally develop from white, rope-like synnemata. Their phialides are flask-shaped, with a swollen base and narrow neck. Most species produce only one neck from the terminal phialides. Some species, e.g., *P. gracilis*, *P. gracilioides*, *P. myrmicarum* and *P. orthopterorum* produce 1–4 necks per phialides. Their conidia are cylindrical or ellipsoid or fusiform. Some species, e.g., *P. orthopterorum* and *P. yodhathaii* have both cylindrical and fusiform forms of conidia (Mongkolsamrit et al., 2019).

Sung et al. (2007a) have concluded that multi-gene phylogeny gave more deeper understanding of phylogenetic relationships of *Cordyceps* and Clavitipitaceae than that of single gene. Recently, the combined LSU-TEF1-α-RPB1 datasets (Mongkolsamrit et al., 2019), combined SSU-LSU-TEF-RPB2 datasets (Ban et al., 2015), and combined SSU-LSU-TEF1-α-RPB1-RPB2 datasets (Quandt et al., 2014; Sanjuan et al., 2015) were allowed for intraspecific and intergeneric identification within Ophiocordycipitaceae. However, individual gene phylogenies are rarely utilized for identification of species in *Paraisaria*.

### Key to the Accepted Species in *Paraisaria*

(1) Host belong to Hymenoptera……………………*P. myrmicarum*
(1) Host not belong to Hymenoptera…………………………………….2
(2) Fertile part colorless…………………………………………………………3
(2) Fertile part pigmented……………………………………………………..4
(3) Fertile part constrict at the center………………………….***P. arcta***
(3) Fertile part is not constricted at the center………………………5
(4) Stromata gregarious…………………………………………………………6

(4) Stromata solitary……………………………………………………………..7
(5) Stromata branched………………………………………….*P. tettigonia*
(5) Stromata unbranched……………………………………………..***P. alba***
(6) Stromata equal or shorter than 20 mm………..*P. blattarioides*
(6) Stromata longer than 20 mm……………………………………………8
(7) Attack nymph stage of host……………………………………………..9
(7) Attack larva stage of host……………………………………………….10
(8) Fertile part reddish brown…………………………….*P. amazonica*
(8) Fertile part grayish yellow……………………………..*P. yodhathaii*
(9) Stromata long, 120 mm…………………………………*P. heteropoda*
(9) Stromata short, 10–45 mm………………………*P. orthopterorum*
(10) Host belong to Coleoptera……………………………………………..11
(10) Host belong to other order of insect………………………………12
(11) Stromata equal or shorter than 14.5 mm………………***P. rosea***
(11) Stromata longer than 14.5 mm………………………………………13
(12) Pathogenic on larva of Diptera (*Coenomyia*). ……………………………………………………………………*P. coenomyiae*
(12) Pathogenic on larva of Lepidoptera (*Hepialidae*) …………………………………………………………………………..*P. gracilis*
(13) Phialides solitary or in whorls of 2–3, with one neck………………………………………………………..*P. phuwiangensis*
(13) Phialides sympodially proliferating, with 1–4 necks…………………………………………………………….*P. gracilioides*

## Data Availability Statement

The datasets presented in this study can be found in online repositories. The names of the repository/repositories and accession number(s) can be found below: https://www.ncbi.nlm.nih.gov/genbank/, MN943843, MN943839, MN929085, MN929078, MN929082, and MN947219; https://www.ncbi.nlm.nih.gov/genbank/, MN943845, MN943841, MN929087, MN929080, and MN947221; https://www.ncbi.nlm.nih.gov/genbank/, MN943844, MN943840, MN929086, MN929079, MN929083, and MN947220.

## Author Contributions

D-PW, DW, and SK: conceptualization. D-PW: data curation. D-PW and DW: formal analysis, methodology, and writing – original draft. SL, ST, and SK: funding acquisition. D-PW and DW: investigation. ST and SK: project administration. KH, J-CX, and PM: supervision. CT-a, AE, SM, ST, SK, KH, J-CX, PM, NS, and SL: writing – review and editing. All authors: contributed to the article and approved the submitted version.

## Conflict of Interest

The authors declare that the research was conducted in the absence of any commercial or financial relationships that could be construed as a potential conflict of interest.

## Footnotes

1 http://mafft.cbrc.jp/alignment/server

2 https://www.phylo.org/portal2/home.action

3 http://purl.org/phylo/treebase/phylows/study/TB2:S25664
